# Postictal Psychosis: Case Report and Literature Review

**DOI:** 10.1155/2023/7960227

**Published:** 2023-03-16

**Authors:** Joana Regala, João Lourenço, Francisco Moniz-Pereira, António Bento

**Affiliations:** ^1^Department of Psychiatry, Psychiatric Hospital Centre of Lisbon (Hospital Júlio de Matos), Lisbon, Portugal; ^2^Department of Neurology, Central Lisbon University Hospital Centre (Hospital Santo António dos Capuchos), Lisbon, Portugal

## Abstract

Postictal psychosis (PIP) is one of the most common types of psychosis in epileptic patients. By virtue of the paucity of research on PIP, its pathophysiology remains not completely understood. Our case report describes a clinical picture of PIP, characterized by pleomorphic features, with neither Schneider's first*-*rank symptoms nor negative symptoms of schizophrenia, in a longstanding epileptic female patient with a history of nonadherence to antiepileptic treatment and poorly controlled seizures. Additionally, she had previous cognitive impairment and encephalomalacia in the right parietooccipital region as a sequela of a moderate-to-severe traumatic brain injury known to precede the emergence of the epilepsy. In light of our findings, we critically reviewed the current literature on postictal psychoses providing insight into its neurobiological underpinnings.

## 1. Introduction

Patients with a long-standing history of seizures are more susceptible to developing major psychiatric disorders, including epileptic psychosis [1]. Psychotic syndromes associated with epilepsy are most seen in patients with longstanding epilepsy with a pharmacoresistant profile or in epileptic patients who are nonadherent to treatment [2] and are normally defined based on their chronological relation to the ictal events [3]. In ictal psychosis (IP), the psychotic symptoms occur during a seizure or epileptic status. Therefore, electroencephalographic documentation of concomitant epileptic activity during the psychotic episode is mandatory for the diagnosis. It manifests during some types of partial seizures, as brief hallucinations of different sensorial modalities, depending on the topographical localization of the ictal focus, or alternatively, it may manifest as immediate precursors to ictal events in the form of epileptic aura [2]. Conversely, interictal psychosis (IIP) is defined as a psychotic episode not temporally related to ictal events, without impairment of consciousness, and might manifest in brief or chronic forms, the latter with a duration of more than 3 months. Phenomenologically, it may be quite similar to primary schizophrenia, yet with better preservation of affect, presenting mood swings and more frequent visual hallucinations. Moreover, IIP has a more benign course and better response to antipsychotics than primary psychosis [4, 5].

Postictal psychosis (PIP), on the other hand, is defined according to Logsdail and Toone's diagnostic criteria as psychotic episodes starting within less than one week after an epileptic seizure, particularly clusters of focal seizures with impaired awareness with or without evolution to generalized tonic-clonic (GTC) seizures [6–8]. After the resolution of the immediate postictal state and before the onset of psychiatric symptoms, the so-called lucid interval occurs which typically lasts up to 72 hours [7, 8]. The presence of a lucid interval allows an accurate differential diagnosis between PIP and postictal confusion [8]. However, the lucid interval may be very short and hence become unnoticed. A hypomanic state often precedes the emergence of psychotic symptoms, usually accompanied by severe anxiety, derealization, insomnia, and cognitive dysfunction [9, 10]. An appropriate and timely therapeutic intervention during this prodromal phase can prevent the subsequent transition into full-blown psychosis [7]. The psychotic symptoms of the nuclear PIP are typically pleomorphic, involving a range of psychotic symptoms, namely auditory, olfactory, gustatory and/or visceral hallucinations, paranoid ideation, mystical delusions, and thought disturbances. Moreover, nuclear PIP tends to be affect-laden and accompanied by some degree of disorientation in clear consciousness [5, 7, 8, 11]. Logsdail and Toone defined the duration of PIP as ranging between 15 hours and two months [6]. Considering accumulated data, PIP typically lasts less than one week and rarely longer than two weeks, with a mean duration of 70 hours [7, 8]. According to the current International League Against Epilepsy guidelines, when the duration of a postictal psychotic episode extends beyond one month, the diagnosis should rather be considered as IIP.

## 2. Case Presentation

**Figure 1:**
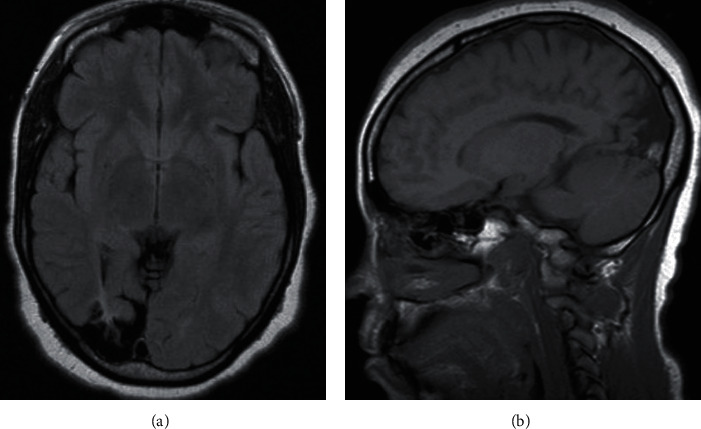
Right cortico-subcortical parietooccipital lesion of encephalomalacia on axial (a) and parasagittal (b) planes of MRI.

A 44-year-old female patient, without a personal or family history of psychiatric illness, was admitted to a psychiatric ward for a psychotic episode which has started three days before, characterized by perplexity, self-referent and mystical delusions, irritability, disorganized behavior, and aggressiveness, that had emerged shortly after a cluster of nocturnal GTC seizures. Neither Schneider's first*-*rank symptoms nor negative symptoms of schizophrenia were present. Additionally, some degree of mental confusion with temporal disorientation, as well as sustained, divided attention and memory deficits were noticed during psychiatric hospitalization. Past medical history was relevant for epilepsy since her early thirties, which had developed after a moderate-to-severe traumatic brain injury (TBI). She did not regularly attend follow-up neurology appointments and had poor adherence to antiepileptic treatment. Ancillary tests undergone during a previous hospitalization in a neurology ward (seven years before the hospitalization in psychiatry) had documented a focal slowing activity in the left frontotemporal topography on electroencephalogram (EEG) and a right cortico-subcortical parietooccipital lesion of encephalomalacia (Figure 1) on magnetic resonance imaging (MRI). Additionally, she presented an amnestic mild cognitive impairment, possibly secondary to both the poorly controlled epilepsy and the prior TBI.

During the hospitalization in the psychiatric ward for the index psychotic episode, haematological and biochemical screening, thyroid function, folic acid, and cyanocobalamin were normal, serologies for VDRL, HIV, and B and C hepatitis were negative, and valproic acid levels were subtherapeutic. She underwent a computed tomography (CT) scan, which did not reveal additional neuroimaging findings to those evidenced by the previous MRI, and an EEG, which was unremarkable.

Psychotic symptoms subsided in the first 36 hours after admission upon treatment with aripiprazole 10 mg/day, valproic acid 1000 mg/day, and diazepam 10 mg taken as required. Aripiprazole was tapered off in the following months. There was no recrudescence of psychotic symptoms with the improvement of seizure control in the outpatient follow-up of neurology appointments.

## 3. Discussion

Regarding the approach to the evaluation of an epileptic patient with an acute psychosis emerging during the postictal period, performing an EEG during the psychotic symptoms is relevant to rule out an ongoing epileptiform activity or a nonconvulsive status epilepticus, which could account for the diagnosis of ictal psychosis. Moreover, a thorough anamnesis is crucial. If there is a history of previous psychotic episodes without temporal relation to ictal events, a diagnosis of IIP should be rather considered. A recent change in the epileptic regimen should raise the suspicion of a psychosis secondary to anticonvulsant drugs or a psychosis secondary to forced normalization/alternate psychosis. Medication-induced psychosis may be caused by some anticonvulsants, namely, levetiracetam, topiramate, zonisamide, ethosuximide, vigabatrin, and tiagabine. The diagnosis of psychosis secondary to forced normalization implies that the onset of psychotic symptoms occurs alongside the presence of at least one out of the two nuclear diagnostic criteria, namely, the reduction in the epileptiform activity on the EEG or the absence of seizures for more than one week. In case that only the latter nuclear diagnostic criterion is present, a further support diagnostic criterion is required, such as a recent change of antiepileptic regimen treatment or previous similar behavioral disturbances suggestive of psychotic symptoms reported by collateral informants [12]. If there is a history of a TBI in the last few weeks, psychotic symptoms must be distinguished from a posttraumatic confusional state, which may manifest during the TBI recovery as fluctuating disorientation, cognitive impairment, psychomotor restlessness, sleep-wake disturbance, and transient psychotic symptoms [13]. Another diagnostic challenge is to what extent psychiatrists may ascribe this clinical condition to an epilepsy-related psychosis rather than to a coincident primary psychosis.

In our patient, the diagnosis of PIP was made upon clinical grounds, considering the emergence of a psychotic episode in a patient with longstanding epilepsy, poor control of seizures, a chronological relation between a cluster of seizures, and the onset of psychotic symptoms with pleomorphic features, yet with neither Schneider's first rank symptoms nor negative symptoms typical of primary schizophrenia, with a rapid resolution, in the absence of a previous personal history of psychotic episodes without temporal relation to ictal events and without having a recent antiepileptic regimen change. The normal EEG undergone during the PIP episode ruled out a possible ongoing intermittent subclinical seizure activity/nonconvulsive epileptic status, making unlikely the diagnosis of ictal or peri-ictal psychosis. Since it is not possible to detect an ongoing deep focal limbic seizure through a classic EEG, which would require an intracranial EEG, the diagnosis of PIP is always presumptive.

Several risk factors for PIP have been posited, namely, bilateral or widespread neural network dysfunction, that may stem from various forms of brain damage, such as encephalitis or TBI, bilateral independent interictal and ictal foci, impaired intellectual function, GTC seizures or focal seizures secondarily generalized, epilepsy with more than 10 years of duration, prior PIP, a personal history of primary psychosis, and a familial history of psychiatric illness [14–16]. Postictal psychosis is more often associated with focal epilepsy, especially temporal and frontal lobe epilepsies, than with idiopathic generalized epilepsy [8]. No clear pattern has been clarified regarding the hemisphere lateralization of the ictal focus. Amongst the aforementioned risk factors for PIP, the most consistent is the evidence of prior extensive brain damage, bilateral interictal epileptiform activity, and slowing on the EEG [14]. In regard to brain damage, it is noteworthy that moderate-to-severe TBI results in two forms of primary brain injury, namely, focal brain lesion and diffuse axonal injury. The latter might contribute to cognitive impairment through large-scale neural network structural dysconnectivity. Eventually, secondary brain injury ensues through several mechanisms, such as loss of cerebral autoregulation, excitotoxicity, mitochondrial dysfunction, oxidative stress, neuroinflammation, and neurodegeneration. The neurodegenerative processes, which occur over several years, can bring about atrophy in brain regions that are far removed from the primary brain injury as it tracks the Wallerian degeneration of damaged axons, disrupting the long-distance connection between brain regions within large-scale intrinsic connectivity networks [17].

The focal brain lesion of encephalomalacia in the right parietooccipital region of our patient is a sequela of the prior TBI known from the anamnesis to precede the emergence of the epilepsy. A previous EEG of our patient had documented a focal slowing in the contralateral hemisphere to the lesion of encephalomalacia, reflecting bilateral cortical damage, which may be explained by secondary brain injury mechanisms. Therefore, we hypothesize that both the primary brain injury, comprising the focal lesion of encephalomalacia and a putative large-scale neural network structural dysconnectivity derived from diffuse axonal injury, and the further secondary brain injury, which ensued after a timespan of several years after TBI, alongside the longstanding and poorly controlled epilepsy, rendered the brain more vulnerable to psychosis.

Notably, one of the foremost neurobiological mechanisms which have been proposed is a “rebound” cortical activation phenomenon after the initial period of postictal suppression [14] from the observed cortical hyperperfusion as assessed by single-photon emission computed tomography (SPECT) scan, particularly in the temporal and frontal lobes [18–20]. For that reason, we further hypothesize that the cerebral cortical volume loss derived from the focal lesion of encephalomalacia (primary brain injury) alongside the further secondary brain injury might have been crucial to the pathophysiology of PIP in our patient by means of a failure of cortical inhibition, hence contributing to the “rebound” cortical activation phenomenon following postictal suppression.

Additional neurobiological mechanisms have been hypothesized, namely, molecular and cellular alterations of epileptogenesis (kindling phenomena), as well as transient postictal neurochemical and neurophysiological dysfunction of limbic structures, including the occurrence of continual epileptiform activity in the cingulate and medial temporal cortices [8, 21].

Furthermore, the absence of a personal or familial history of psychiatric illnesses in our patient may be a cue that factors related to a heightened vulnerability to epilepsy play a more significant role than genetic factors related to psychosis in the emergence of PIP. In fact, although the relevance of genetic factors related to psychosis is not consistent across different studies on PIP [15], some reports evidence a family history of psychosis, though in the absence of an identified structural brain lesion [22, 23].

PIP is usually self-limited. The mainstay of treatment relies on appropriate seizure control with antiepileptic treatment. It typically responds to benzodiazepines and/or low-dose atypical antipsychotic drugs in the acute phase [8]. In contrast to IIP, no long-term antipsychotic treatment is needed. The short-term prognosis of PPI is often favourable compared to other psychotic syndromes associated with epilepsy, namely IIP. However, in the longitudinal course, half of the PIP cases become recurrent, and from those, around 15-20% may go on to develop chronic IIP, in which long-term antipsychotic treatment is mandatory [14, 16]. PIP is theoretically preventable, provided that epilepsy is properly controlled. Therefore, a multidisciplinary approach involving a good collaboration between psychiatrists and neurologists is highly desirable to provide the best level of clinical care to epileptic patients who manifest PIP.

In conclusion, this case report highlights the paramount relevance of widespread neural network dysfunction alongside longstanding and poorly controlled epilepsy, over genetic factors predisposing to primary psychosis, in the pathophysiology of the PIP. The long-time latency between the onset of posttraumatic epilepsy and the emergence of PIP observed in our case endorses the hypothesis that gradual physiological and structural brain alterations, namely, kindling phenomena and secondary brain injury with cerebral volume loss, contributed to an increasing vulnerability to psychosis, eventually ensuing in a state wherein a cluster of seizures becomes able to elicit a psychotic episode. According to this proposed model, a cluster of seizures would uncover the deficits derived from diffuse brain dysfunction, such as a failure of cortical inhibition in the postictal phase.

Notwithstanding, there is hitherto not sufficient research data to elaborate in depth on the neurobiological mechanisms of PIP. Further research should continue to explore this issue through neurophysiologic studies, such as postictal intracranial EEG, and neuroimaging studies, such as postictal SPECT and neurochemical imaging with positron emission tomography, as well as diffusion tensor MRI to identify the impaired large-scale neural networks.
